# Supplementary material for: Immunogenic Responses Elicited by a Pool of Recombinant Lactiplantibacillus plantarum NC8 Strains Surface-Displaying Diverse African Swine Fever Antigens Administered via Different Immunization Routes in a Mouse Model
Source: Vaccines (Basel). 2025 Aug 25;13(9):897. doi: 10.3390/vaccines13090897 (PMC12474437; doi:10.3390/vaccines13090897)
Supplement: Supplementary file 1 [file vaccines-13-00897-s001.zip › vaccines-3744786-supplementary.pdf]

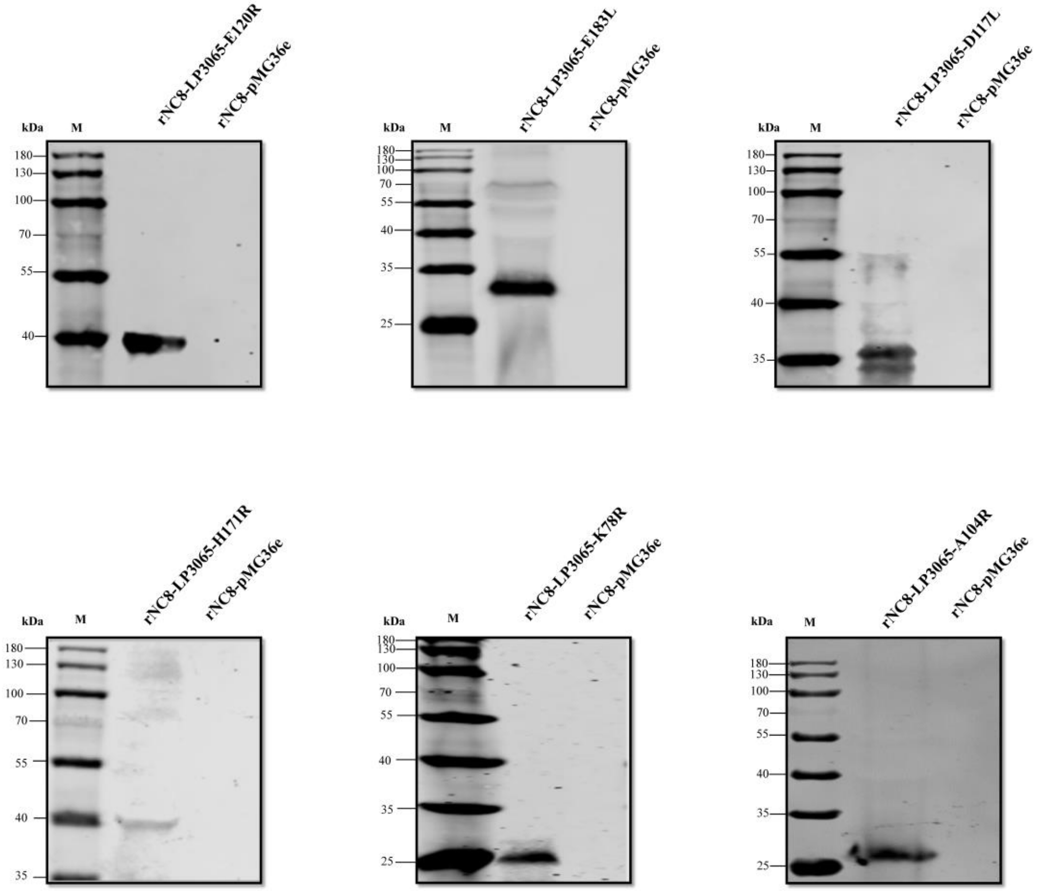

Figure S1. Western blotting analysis of antigen expression in rNC8-LP3065-ASFV-mix

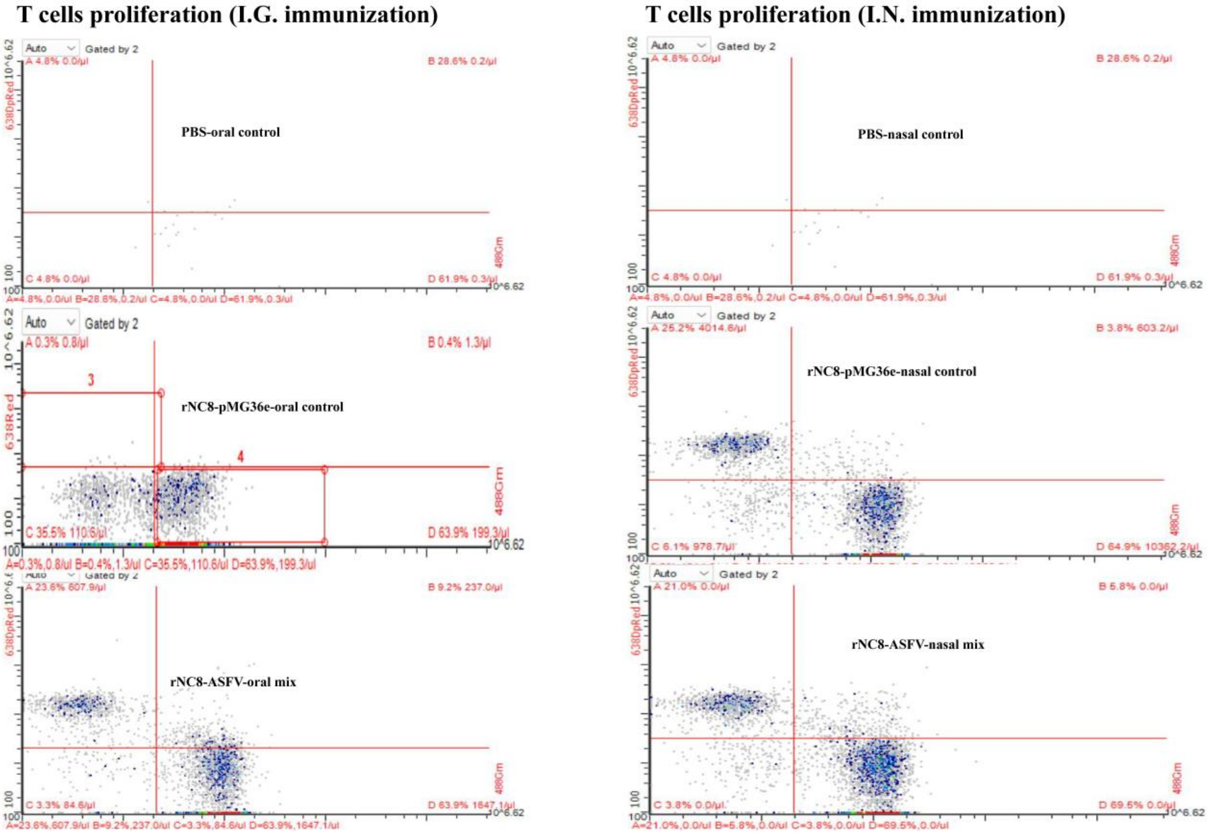

Figure S2. Flow cytometry analysis for detection of CD3CD4^{+} and CD3CD8^{+} T cells
